# Supplementary material for: The Lectin-Like Domain of Thrombomodulin Inhibits β1 Integrin-Dependent Binding of Human Breast Cancer-Derived Cell Lines to Fibronectin
Source: Biomedicines. 2021 Feb 7;9(2):162. doi: 10.3390/biomedicines9020162 (PMC7914543; doi:10.3390/biomedicines9020162)
Supplement: Supplementary file 1 [file biomedicines-09-00162-s001.pdf]

The lectin-like domain of thrombomodulin inhibits β1 integrin-dependent binding of human breast cancer-derived cell lines to fibronectin

Eiji Kawamoto, Nodoka Nago, Takayuki Okamoto, Arong Gaowa, Asami Masui-Ito, Yuichi Akama, Samuel Darkwah, Michael Gyasi Appiah, Phyoe Kyawe Myint, Gideon Obeng, Atsushi Ito, Siqingaowa Caidengbate, Ryo Esumi, Takanori Yamaguchi, Eun Jeong Park, Hiroshi Imai, and Motomu Shimaoka

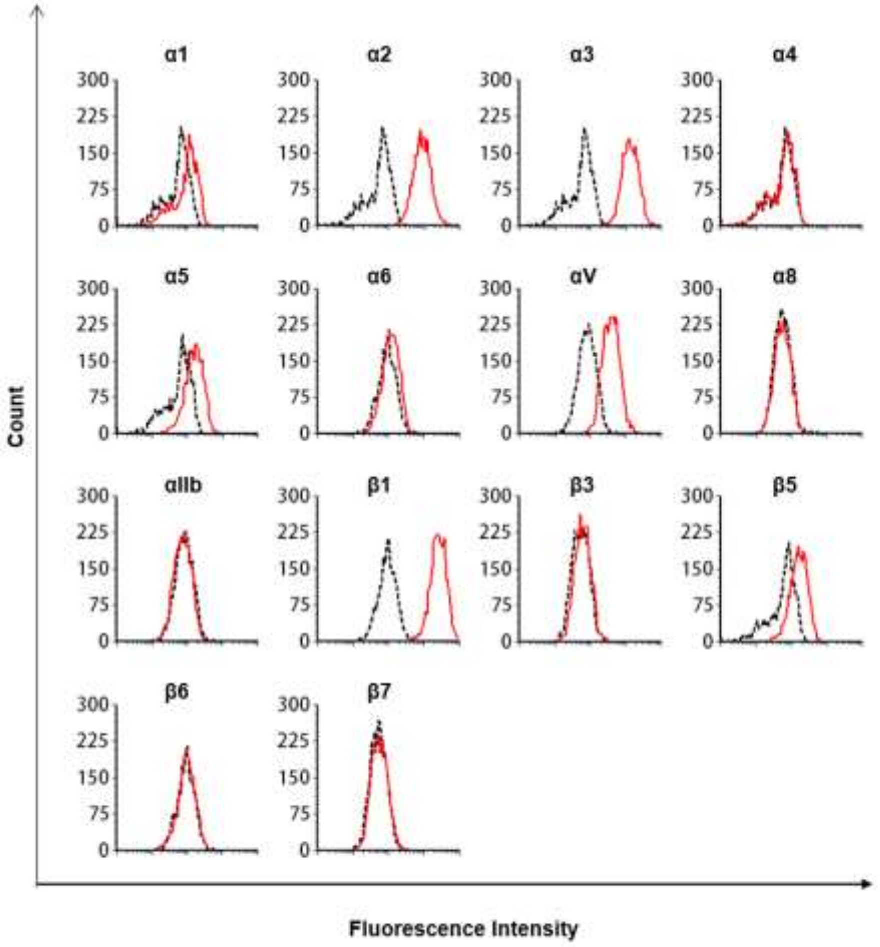

Figure S1. Flow cytometry analysis of integrin expression on MCF-7 cells. Dotted lines represent isotype controls, and solid lines represent integrin expression. The figure is a representative of three experiments with similar results.
